# Supplementary material for: Characterization of the biochemical activity and tumor-promoting role of the dual protein methyltransferase METL-13/METTL13 in Caenorhabditis elegans
Source: PLoS One. 2023 Jun 22;18(6):e0287558. doi: 10.1371/journal.pone.0287558 (PMC10286969; doi:10.1371/journal.pone.0287558)
Supplement: S1 File — (DOCX) [file pone.0287558.s001.docx]

**Characterization of the biochemical activity and tumor-promoting role of the dual protein methyltransferase METL-13/METTL13 in *Caenorhabditis elegans*.**

Melanie L. Engelfriet^1^, Jędrzej M. Małecki^1^, Anna F. Forsberg^1^, Pål Ø. Falnes*^1^ and Rafal Ciosk*^1^.

^1^ Department of Biosciences, Faculty of Mathematics and Natural Sciences, University of Oslo, Oslo, Norway

* Corresponding authors: rafal.ciosk@ibv.uio.no (RC) and pal.falnes@ibv.uio.no (PØF)

**
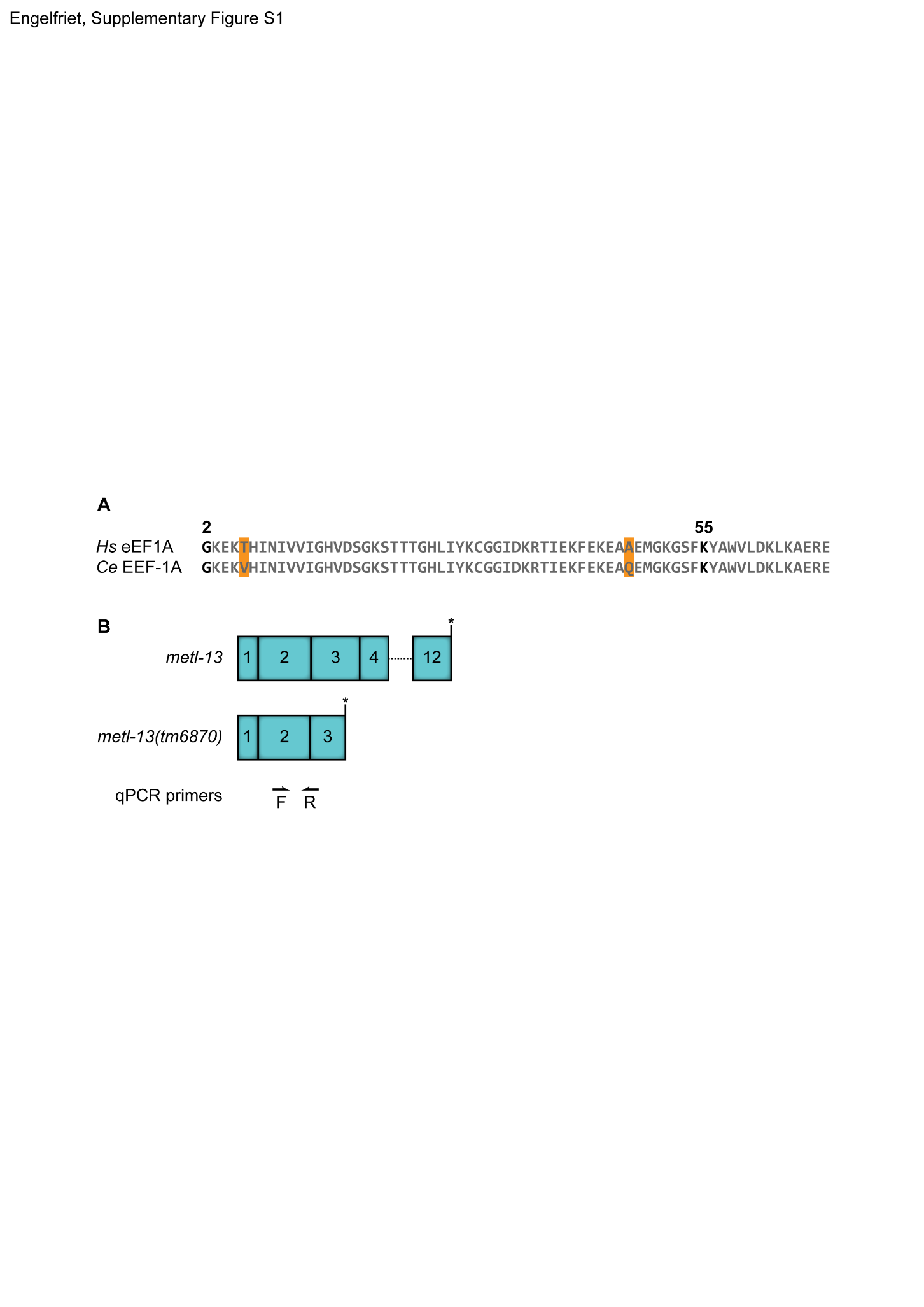
**

**S1 Fig. Analysis of C. elegans METL-13/METTL13 and EEF-1A/eEF1A.**

**A.** Partial alignment of human (Hs) eEF1A and C. elegans (Ce) EEF-1A. EEF-1A is identically encoded by two genes: eef-1A.1 and eef-1A.2. The G2 and K55 residues (in bold) are conserved between eEF1A and EEF-1A. Non-conserved residues are colored orange. **B.** Schematic representation of the C. elegans metl-13 gene (numbered boxes represent the exons, * indicated the stop codon) and the region deleted in the metl-13(tm6870) allele, along with the used qPCR primers (F – forward, R - reverse).


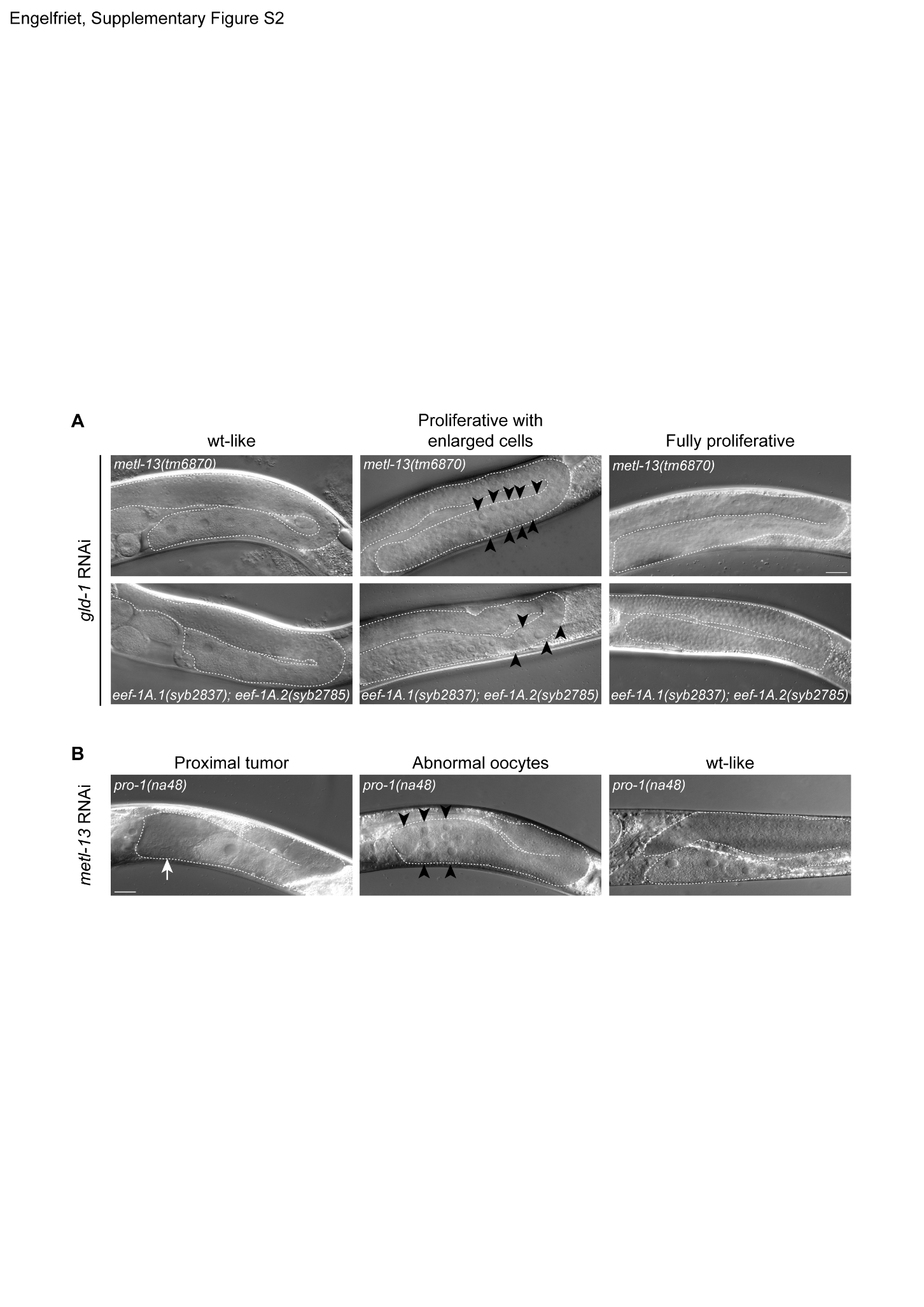


**S2 Fig. Depletion of METL-13 and EEF-1A^K55me2^ reduces the severity of germline tumors**

**A.** DIC micrographs of adult *metl-13(tm6870)* and *eef-1A.1(syb2837); eef-1A.2(syb2785)* animals subjected to *gld-1* RNAi. The gonads are outlined. Three phenotypes were observed after *gld-1* RNAi, we refer to these gonads as ‘fully proliferative’, ‘proliferative with enlarged cells’ (black arrowheads indicate enlarged cells), and ‘wt-like’ respectively. Scale bar = 20 µm. **B.** DIC micrographs of *pro-1(na48)* mutants (grown at the restrictive temperature, 25°C) subjected to *metl-13* RNAi. The gonads are outlined. Three phenotypes were observed: we refer to seemingly normal germlines as ‘wt-like’, germlines containing proliferating cells proximal to the gametes as ‘proximal tumor’ (indicated by a white arrow), and germlines lacking a proximal tumor that instead contain abnormal oocytes as ‘abnormal oocytes’ (indicated by black arrowheads). Scale bar = 20 µm.

**
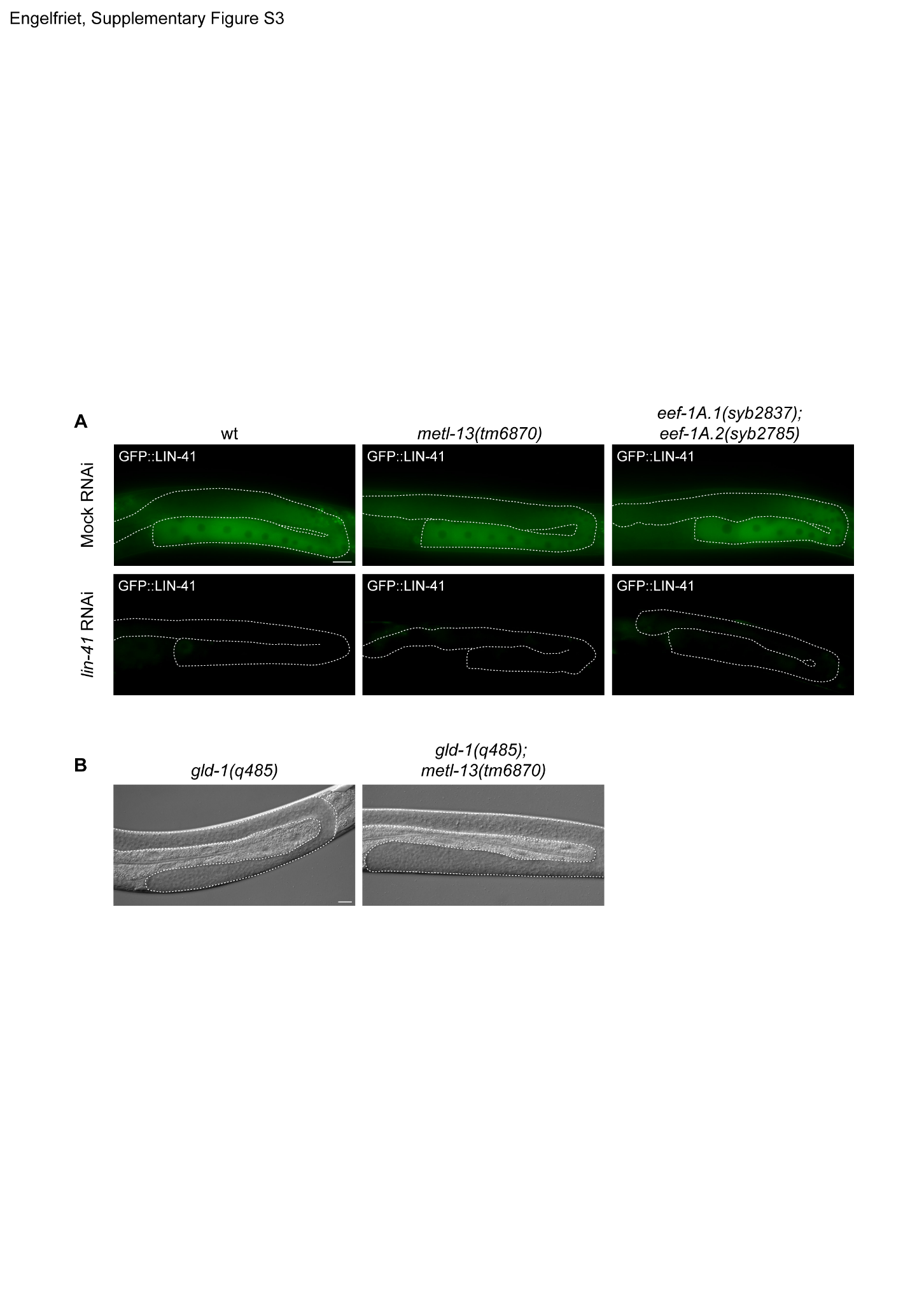
**

**S3 Fig.** **RNAi is as effective in *metl-13* mutants as in wild-type.**

**A.** RNAi-mediated depletion of GFP-tagged LIN-41 (GFP::LIN-41) is comparable between wt, *metl-13(tm6870)*, and *eef-1A.1(syb2837); eef-1A.2(syb2785)* animals. The gonads are outlined. Scale bar = 20 µm. **B.** DIC micrographs of *gld-1(q485)* null mutant and *gld-1(q485); metl-13(tm6870)* double mutant animals. The gonads of both animals contain large tumors consisting of small proliferative cells. The gonads are outlined and the proximal end is indicated with an asterisk. Scale bar = 20 µm.
