# Supplementary material for: Characterization of the biochemical activity and tumor-promoting role of the dual protein methyltransferase METL-13/METTL13 in Caenorhabditis elegans
Source: PLoS One. 2023 Jun 22;18(6):e0287558. doi: 10.1371/journal.pone.0287558 (PMC10286969; doi:10.1371/journal.pone.0287558)
Supplement: S1 Raw images — (PDF) [file pone.0287558.s002.pdf]

Fig 2A

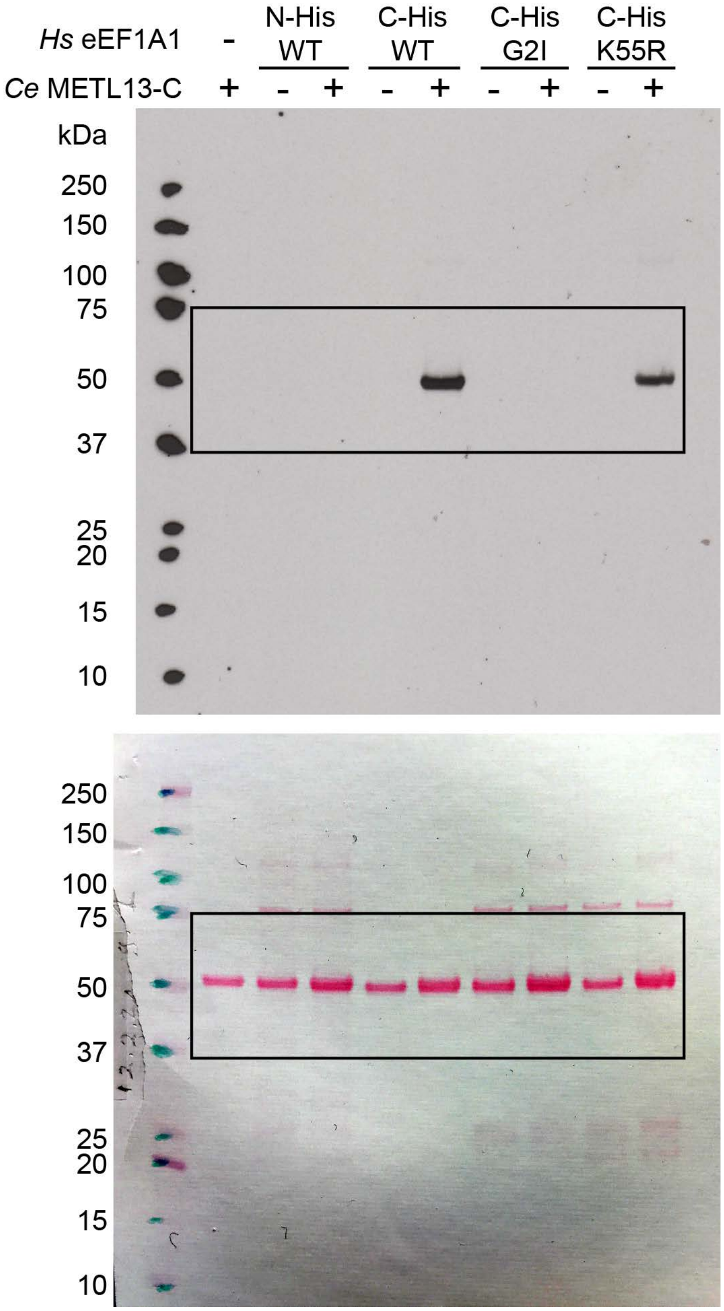

Fig 2A top panel was created from this original image.

Fig 2A bottom panel was created from this original image.

Fig 2B

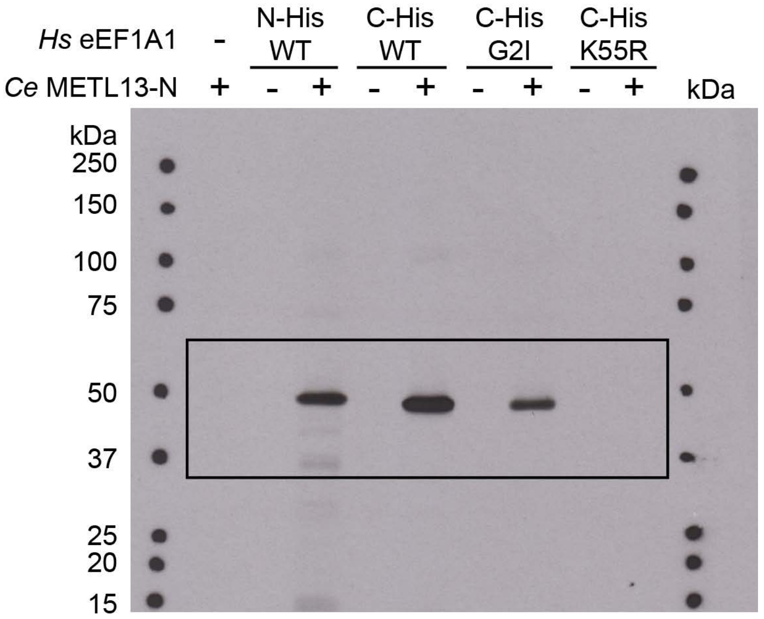

Fig 2B top panel was created from this original image.

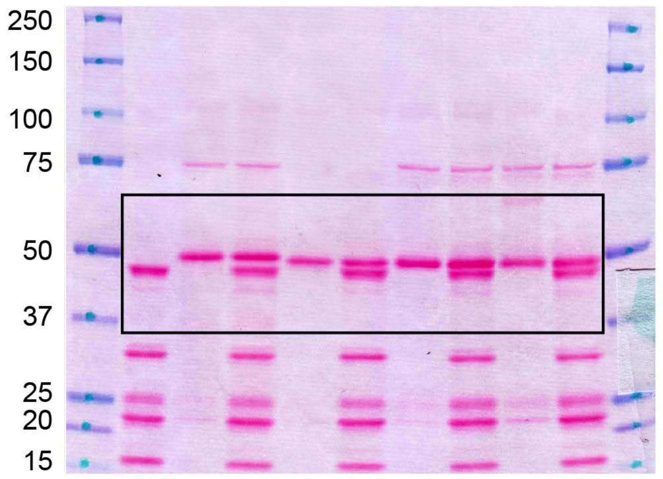

Fig 2B bottom panel was created from this original image.

Fig 3A

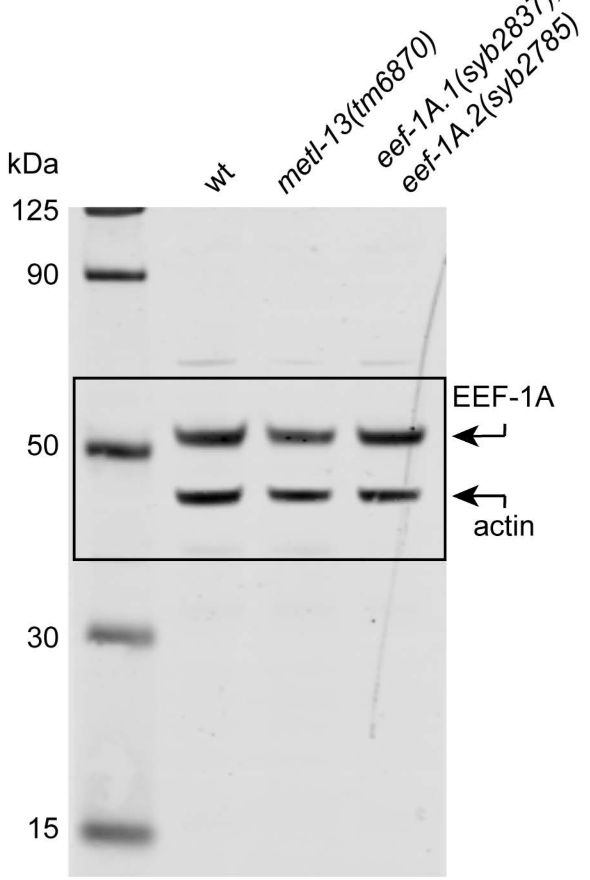

Fig 3A left panel was created from this original image.
